# Are uterine natural killer and plasma cells in infertility patients associated with endometriosis, repeated implantation failure, or recurrent pregnancy loss?

**DOI:** 10.1007/s00404-020-05679-z

**Published:** 2020-07-14

**Authors:** Nadine Freitag, Sarah J. Pour, Tanja N. Fehm, Bettina Toth, Udo R. Markert, Maja Weber, Riku Togawa, Jan-Steffen Kruessel, Dunja M. Baston-Buest, Alexandra P. Bielfeld

**Affiliations:** 1 Department of Obstetrics, Gynecology and REI (UniKiD), Medical Faculty, Medical Center University of Düsseldorf, University Hospital Düsseldorf, Moorenstr. 5, 40225 Düsseldorf, Germany; 2 Department of Obstetrics and Gynecology, Medical Center University of Düsseldorf, Moorenstr. 5, 40225 Düsseldorf, Germany; 3 Gynecological Endocrinology and Reproductive Medicine, Medical University Innsbruck, Innsbruck, Austria; 4 Placenta Lab, Department of Obstetrics, Jena University Hospital, Am Klinikum 1, 07740 Jena, Germany; 5 Department of Gynecological Endocrinology and Fertility Disorders, Karls-Ruprecht University, Heidelberg, Germany

**Keywords:** Chronic endometritis, Endometriosis, Endometrium, Immunotherapy, Soybean oil, Spontaneous abortion

## Abstract

**Purpose:**

Infertility is a debilitating situation that millions of women around the world suffer from, but the causal relationship between infertility and endometriosis is still unclear. We hypothesize that the immune cell populations of uterine natural killer cells (uNK) and plasma cells (PC) which define chronic endometritis could differ in patients with or without endometriosis and therefore be the link to endometriosis-associated infertility.

**Methods:**

Our retrospective study includes 173 patients that underwent an endometrial scratching in the secretory phase of the menstrual cycle and subsequently immunohistochemical examination for uNK cells and PC. Sixty-seven patients were diagnosed with endometriosis, 106 served as the control cohort.

**Results:**

The risk for an elevated number of uNK cells in women with endometriosis is not increased as compared to the control group. Our findings suggest that patients with endometriosis are 1.3 times more likely to have chronic endometritis (CE) as compared to those without and that the treatment with doxycycline might increase pregnancy rates. Endometriosis and an increased number of uNK cells seem to be unrelated.

**Conclusions:**

In contrast to the lately published connection between endometriosis, infertility and increased uNK cells, we could not find any evidence that patients with endometriosis are more prone to elevated uterine uNK cells. Counting of PC in endometrial biopsies might be a new approach in the search of biomarkers for the nonsurgical diagnosis of endometriosis since our findings suggest a connection.

## Introduction

Involuntary childlessness is a disconsolating situation that around 12–15% of couples in the reproductive phase suffer from [1, 2]. The prevalence of infertility in women with endometriosis is 11.6% while women without endometriosis only have a prevalence of 3.4% [3]. The causal relationship between endometriosis and infertility is still unclear, and 4 theories are discussed. The most widespread theory suggests that pelvic inflammations caused by endometriosis leads to pelvic adhesions and engender functionally impaired fallopian tubes, that consequently compromise fertility [4]. Another theory suggests that endometriosis-associated infertility develops due to immunological dysfunctions. Endometriosis is a chronic pelvic inflammatory disease with an increased number of inflammatory cells and mediators in the peritoneal fluid [5]. Therefore, the microenvironment of the eutopic endometrium might be altered too, and accordingly, the successful development of a pregnancy may be affected.

uNK cells are very important for a successful pregnancy, but could also be harmful if dysregulated in number and/or function. uNK cells represent 70% of the leukocytes in the endometrium during the secretory phase of the menstrual cycle and the time of embryo implantation [6, 7]. Once activated, uNK cells can produce angiogenic factors, such as vascular endothelial growth factor (VEGF) and hence, promote spiral artery remodeling which supports the implantation of the trophopblast [8]. Through secreting cytokines, uNK cells may create a pro-invasive environment in the endometrium and promote the migration and invasion of the trophoblast [9]. However, a study with 61 patients revealed that patients with an increased number of uNK cells are at a greater risk for infertility disorders [10]. Furthermore, the developmental stage of uNK cells, and; therefore, their functions seem to impinge upon a normal pregnancy. Patients with endometriosis seem to have an abnormal maturation of uNK cells with lower levels of endometrial stem cell factor, that might lead to endometriosis-associated infertility. In vitro, stem cell factor can restore uNK cell maturation [11]. In conclusion, uNK cells seem to play an important role in the development of pregnancy. However, if they differ in their number and/or function, uNK cells may become a serious threat to implantation and pregnancy. We hypothesize that beside possible effects on the maturation process, endometriosis also influences the number and function of uNK cells.

A new therapeutic approach to treat an increased number of uNK cells is the administration of an intravenous lipid infusion [12]. This fat emulsion contains soybean oil, glycerin, and egg phospholipids, and is commonly used as part of an intravenous nutrition for patients who are unable to be nourished orally [13]. In vitro studies have revealed that intravenous lipid infusion can suppress the NK cells´ cytotoxicity in the periphery [14]. In vivo, both ongoing pregnancy rate (OPR) and live birth rate (LBR) in women with unexplained recurrent pregnancy loss (RPL) and elevated peripheral NK activity may be improved by lipid infusion [12, 15]. Nevertheless, the discussion on potential molecular and cellular effects on the establishment of the pregnancy is still controversial [12, 16]. In our study, we analyzed the effect of the lipid infusion on elevated uNK cells and pregnancy rate in women with endometriosis, repeated implantation failure (RIF) or RPL.

Another cell population in the endometrium being associated with endometriosis and infertility are PC. A histopathological determination of PC in the endometrium verifies CE, which is often asymptomatically, and; therefore, not detectable in the anamnesis in the clinical routine. CE usually progresses subclinically or with mild symptoms including abnormal uterine bleeding or menorrhagia [17]. CE is a persistent condition of local endometrial inflammation and its reported incidence is around 9% with an increase in infertile women [17–19]. The rate of CE has been found as 28% in patients with infertility of unknown etiology [20], 30% in patients with RIF [21, 22], and 27% in patients with RPL [23]. Additionally, a strong correlation between CE and endometriosis has been shown. In more than 50% of the biopsies of the endometriosis patients analyzed in this study a minimum number of 1 PC occurred [24, 25]. Unfortunately, a committed definition regarding the amount of PC in a defined area for the diagnosis of CE is still missing.

The treatment with a broad-spectrum antibiotic, such as doxycycline could effectively cure CE in 75% [22]. In about 25% of the cases, CE remains after the 1st antibiotic treatment [22]. Pregnancy rates subsequent to the treatment are significantly higher in women with a history of RPL who have been cured as compared to those with a persistent CE [22, 26].

## Materials and methods

### Participants of the study

The retrospective study was approved by the ethical committee of the medical faculty of the Heinrich-Heine University, Düsseldorf, Germany for the patients enrolled in Düsseldorf and for the diagnostics in Jena. All women gave their written informed consent. Female participants underwent an endometrial scratching in the secretory phase of the menstrual cycle and subsequently immunohistochemical examination for uNK cells and PC between January 2013 and February 2017. The examination was performed as part of diagnostic measures before assisted reproductive treatment (ART), including scheduled intercourse, insemination, and in vitro techniques or if requested by the patient. RIF was assumed in a patient if there was no occurrence of pregnancy after at least two high quality embryo transfers (or three, if the patient was older than 40 years). RPL was defined as two or more abortions after the 7th week of pregnancy.

In total, 173 infertility patients (> 90% Caucasian) who underwent the diagnostic procedure for uNK cells and PC between January 2013 and February 2017 were incorporated in the study. 67 of them were diagnosed with endometriosis (either anamnestic [dysmenorrhea, dyspareunia, dysuria] or laparoscopic) and 106 without endometriosis served as the control cohort. Patients were treated in either the fertility center UniKiD Düsseldorf, Germany or the Kinderwunschzentrum Heidelberg, Germany. The patients’ age ranged between 26 and 48 years.

### IHC for uNK and plasma cells

The endometrial scratching was performed vaginally with a Pipelle Endometrial Suction Curette (Probet, Gynmed GmbH, Germany). The samples were fixed in 4% formaldehyde and sent to the Placenta Lab at the Jena University Hospital, Germany, where it was immunohistochemically stained for CD56-postive uNK cells and/or CD138-positive PC. A number of at least 300 uNK cells or at least 5 PC per mm^2^ were defined as elevated according to the publications of Kuon et al. [16] and Bayer-Garner et al. [27, 28].

### Therapeutic approach

Elevated uNK cells were treated with an intravenous infusion of *Intralipid* in the course of the ART treatment (8% Intralipid in 250 ml NaCl: 1st infusion on the day of ovum pickup or the day of the embryo transfer in frozen embryo transfer cycles, 2nd infusion after the positive pregnancy test, 3rd and following infusions every 2 weeks until 12th week of pregnancy), elevated PC were treated with a course of doxycycline (200 mg per day over 21 days) prior to treatment. In most cases, the reduction in PC after the doxycycline treatment was determined by a 2nd scratching and analysis at the University Hospital Jena. In addition, the following parameters were examined: patient age, day of the cycle biopsy was taken, body mass index (BMI), smoking status, obstetrical history, presence of RIF or RPL, presence and severity of endometriosis according to the classification of endometriosis by the Society of Obstetricians and Gynaecologists of Canada [29]. Pregnancy was assumed when the blood level of human chorionic gonadotropin (β-hCG) was higher than 20 international units (IU) per liter 14 days after fertilization.

### Statistics analysis

The risk for elevated uNK cells or PC was compared using Odd’s ratio (OR) calculations. Life style factors between different groups were contrasted using the two-tailed Student’s *t* test. Levene’s test assessed the equality of variances within the groups. Fisher exact test was used to compare the presence of endometriosis, elevated uNK cells and/or PC, and occurrence of RPL/RIF (analyses were performed using Microsoft Excel 2010). A level of *P* ≥ 0.05 was considered as the limit for significance.

## Theory

In this study, we aimed to analyze whether infertility patients with endometriosis have a higher prevalence for increased uNK or PC in the endometrium and if their modulation with an intravenous lipid infusion or doxycycline can increase pregnancy rates.

## Results

171 patients underwent analysis for eutopic uNK cells. 43 (25.1%) patients had an elevated number of uNK cells with an average number of 385.8 uNK cells per mm^2^ (± 117.8). 53.5% of the patients with an increased level of uNK cells reported an abortion prior to the examination. 16 of the patients with elevated uNK cells suffered from endometriosis, 17 from RIF, and 16 from RPL (Table 1). Out of 16 patients with both endometriosis and elevated uNK cells, 9 (56.3%) had at least one abortion prior to the examination. OR calculations did not indicate an effect of endometriosis or increased levels of uNK on an abortion prior to the examination [OR = 1.0776/1.3952; 95% confidence interval (CI) 0.5782–2.0084/0.6924–2.8112; *P* = 0.8140/0.8140].

**Table 1:** Distribution of uNK cells in all patients

uNKs/mm^2^	No. of patients	No. of patients with endometriosis	No. of patients with RIF	No. of patients with RPL
< 100	52	18	22	11
100–299	76	31	35	12
> 299	43	16	17	11

	No. of patients	> 299 uNKs/mm^2^	No. of patients with an embryo transfer in the following cycle	All documented live births
Endometriosis	67	16	4	3
RIF	74	16	10	2
RPL	34	11	1	1
No endometriosis, RIF or RPL	53	11	5	1

Indicated is the no. of patients diagnosed for uNK cells and subdivided in patients suffering from endometriosis, RIF or RPL. Furthermore, the follow-up of patients treated with soybean oil infusion is shown

Sixty-seven patients with endometriosis were compared with 106 patients without endometriosis. In the endometriosis group, 23.9% had an elevated number of uNK cells in the endometrium. In the control group, 25.5% of the women had an increased number of uNK cells. Odd’s ratio calculations showed that the frequency of having an elevated number of uNK cells does not differ for women with endometriosis as compared to those without (OR = 0.9179; 95% confidence interval 0.4506–1.8700; *P* = 0.8135) (Table 1).

Thirty-five patients (numbers in parentheses represent patients with endometriosis: 9) received an intravenous lipid infusion in order to treat elevated levels of uNK cells in the endometrium, of which 17 (3) received a transfer of at least one high quality embryo. The remaining 18 patients (6) did not receive an embryo transfer due to absence of a high-quality embryo, delayed transfer to a subsequent cycle or different treatment options such as insemination or scheduled intercourse. 5 (2) out of the 17 (3) patients with a transfer of a high-quality embryo following ART and one endometriosis-patient after scheduled intercourse had a live birth subsequent to the treatment (see Table 1). In the group of 5 (2) patients who had a live birth after intralipid therapy, 3 (1) underwent in vitro fertilization (IVF), 2 (0) underwent intracytoplasmic sperm injection (ICSI), and one had scheduled intercourse. Owing to the limited number of patients in the individual subgroups, a statistical analysis did not seem to be appropriate here.

Of all patients, 43 showed elevated uNK cells. Five patients with elevated uNK cells suffered simultaneously from endometriosis and RIF, six patients with elevated uNK suffered simultaneously from endometriosis, and RPL. In 11 patients with elevated uNK neither endometriosis, nor RIF or RPL have been reported (Table 1).

112 patients underwent examination for eutopic PC. 13 patients (11.6%) showed an elevated number of PC with an average number of 15.7 PC per mm^2^ (± 17.7). Four of those patients with an increased number of PC (three with endometriosis) reported an abortion prior to the examination. Eight of the patients with increased PC suffered from endometriosis, six patients from RIF and 2 from RPL (Table 2).

**Table 2:** Distribution of PC in all patients

PC/mm^2^	No. of patients	No. of patients with endometriosis	No. patients with RIF	No. of patients with RPL
0	79	42	37	20
1–4	16	10	6	6
> 4	13	8	6	2

	No. of patients	> 4PC/mm^2^	No. of patients with an embryo transfer in the following cycle	Documented live births
Endometriosis	67	8	3	1
RIF	74	6	5	1
RPL	34	2	0	0
No endometriosis, RIF or RPL	53	3	1	1

Indicated is the no. of patients diagnosed for PC and subdivided in patients suffering from endometriosis, RIF or RPL. Furthermore, the follow-up of patients treated with doxycycline is shown

In the endometriosis group (62 patients), 12.9% of the patients had an increased number of PC vs. 10% in the control group (50 patients). The risk for an elevated level of PC and accordingly for the presence of CE was 1.3 times higher in women with endometriosis. However, the result was not significant (OR = 1.333; 95% confidence interval = 0.4075–4.3624; *P* = 0.6343). In order to cure CE, six patients (in parentheses the number of patients with endometriosis): (5) were treated with doxycycline [200 mg/day] for 3 weeks. 5 (4) patients reported previous pregnancies, 2 (2) of them had an abortion after the 12th week of pregnancy and 1 (1) live birth occurred before. The remaining 4 (3) patients with CE reported no previous pregnancies. Within 10 months after the treatment, 7 (4) patients received a transfer of at least one high quality embryo, and 2 (1) patients had a live birth. In total, the live birth rate could be improved from one out of nine before to two out of seven patients after the treatment with doxycycline (Table 2).

Of all patients, 13 showed elevated PCs. Four patients with elevated PCs suffered simultaneously from endometriosis and RIF. Two patients with elevated PCs suffered simultaneously from endometriosis and RPL. Three patients with elevated PCs suffered from neither endometriosis nor RIF or RPL (Table 2).

In total, 74 patients were suffering from RIF, 16 of them had elevated uNK cells (with two subsequent live births), and 6 had elevated PC (with one subsequent live birth). Thirty-four patients suffered from RPL, 11 of them had elevated uNK cells (with one subsequent live birth), and two had elevated PCs. OR calculations did not indicate that elevated uNK or PC could be a risk factor for RIF/RPL (OR = 1.0336/0.6085; 95% CI 0.5065–2.1093/0.1829–2.0243; *P* = 0.9277/*P* = 0.4179).

The BMI of patients with elevated uNK cells (22.4 kg/m^2^ ± 3.44) and PC (21.6 kg/m^2^ ± 7.2) did not differ from those with normal uNK cells and PC (22.9 kg/m^2^ ± 4.9) counts (*P* ≈ 0.33).

## Discussion

The intensive research on biomarkers for endometriosis has not yet been successful and laparoscopic surgery still remains the gold standard for diagnosis [30]. Endometriosis is considered an inflammatory disease and evidence for an altered distribution of immune cells in the uterine cavity has frequently been reported [31–36]. CE and endometriosis are both inflammatory diseases with an unexplained pathogenesis causing symptoms like pain and infertility. Takebayashi et al. showed a significant association between endometriosis and CE in a group of 71 patients [24]. Although in our group of mostly Caucasian patients with endometriosis, 12.9% were diagnosed with CE, the prevalence of CE in the Japanese endometriosis group of Takebayashi et al. reached 52.94%. The striking difference in the prevalence of CE might also be due to the method of acquisition of endometrial samples: Takebayashi et al. used the endometrial specimens from patients who underwent hysterectomy due to gynecological diseases. Our samples were generated from endometrial scratchings in women during ART treatment. Furthermore, different cutoff values for PC diagnosis were applied. Whereas our group defined > 4 PC cells per mm^2^ as a marker for CE, Takebayashi et al. postulated CE as multiple PCs in ten nonoverlapping random fields of endometrial stroma [24]. Nonetheless, samples from both studies were immunostained with anti-CD138 antibody and PC were subsequently counted under a light microscope [24]. Another recent study including 156 patients found a statistically higher prevalence of CE in patients with endometriosis. They also compared two methods hysteroscopy vs. immunostaining—and found a significant connection of CE and endometriosis in both procedures. Pictures photographed during hysteroscopy revealed 42.3% vs. 15.4% occurrence of CE for patients with endometriosis vs. without while immunostained samples for CD138 collected after hysterectomy of the same patients showed 38.5% vs. 14.1% prevalence of CE [25].

Analyzing uterine PCs might be a new approach in the search of biomarkers for endometriosis. The elevated number of PCs in endometriosis patients might result from a bacterial infection due to a dysregulated retrograde menstruation. However, large-scaled studies are necessary to confirm the correlation between endometriosis and CE and more research is required to understand the potential causal connection between both diseases.

In our study, the overall prevalence of CE was 11.6%. Other studies found higher prevalence rates of 30.3% [21], 33.7% [37] and 66% with more than 250 patients included [22]. In comparison with the other studies, our endometrial samples were generated from endometrial biopsies and not from hysterectomy due to a benign gynecological disease. The lower prevalence of CE might result from that difference. The number of patients treated with doxycycline (*n* = 9) in our study is limited, but supports our results. A large study, including 421 patients with RIF published in June 2017 compared the LBR in patients after successful treatment for CE with controls. They found significantly higher birth rates in the treated group, suggesting oral antibiotic treatment for women suffering from RIF as a new therapeutic option [37]. Another retrospective study investigated the outcome of embryo transfers in patients suffering from RIF and CE. They found an improvement of the LBR after antibiotic treatment in fresh embryo transfers [22]. Alongside with testing for and curing the occurrence of uterine PC, uNK cells have been associated with infertility and endometriosis [38], but the negative impact on the development of pregnancy is diverse [9–11, 39–42]. However, increased levels of uNK cells are more and more frequently treated with intravenous lipid infusions [12]. In vitro studies revealed that intravenous lipid infusions can suppress peripheral blood NK cytotoxicity [14]. In our study, we found that the treatment with intravenous lipid infusions tends to increase pregnancy rates in all groups investigated. This observation is due to a limited number of patients only descriptive though. Even though not associated with uNK cells yet, the idea of intravenous lipid infusion therapy for RPL already existed in 1994. Within a double-blinded randomized controlled trial in humans’ intravenous lipid infusions versus trophoblast membrane vesicles was applied in patients with RPL. The prediction made from these data was tested in a mouse model of RPL and intravenous lipid infusions in small doses prevented abortions in mice [43].

In 2012, a study examined the coherence between immunotherapy and LBR in cases with elevated antiphospholipid antibodies or increased peripheral blood NK cells. Intravenous lipid infusions seemed to be a successful therapeutic option for women with reproductive problems and increased peripheral blood NK cells [44]. Check et al. stated contradicting results when they compared two groups of patients at age 40–42 years with a history of RIF or RPL. The study was stopped after the first ten matched cycles because the group of treated patients did not show any pregnancies while the untreated group had 40% clinical PR and a 30% LBR. The authors suggested that the therapy with intravenous lipid infusions in this age group might rather be harmful than beneficial [45]. In line with those results, an Egyptian group compared 296 women with RPL and elevated peripheral blood NKs with a treatment of either intravenous lipid infusions (2 ml diluted at 20% in 250 ml saline) or saline (250 ml) infusion alone applied only once on the day of oocyte retrieval. The difference between both groups was not significant and; therefore, intravenous lipid infusions supplementation did not increase biochemical pregnancies [12]. Within a study, including 40 RPL patients with NK disorders treated with lipid infusions starting at positive pregnancy test, every 2 weeks until 12 + 0 weeks of gestation prepregnancy immune diagnostics differed significantly with higher levels of peripheral NK (% and /µl) in RPL patients who miscarried again as compared to controls [40].

Even though there is no large-scale study yet proving the positive effect of intravenous lipid infusion pregnancy outcomes, the publications reveal evidence of a positive effect. Contradicting the lately published connection between endometriosis, infertility, and increased uNK cells [38], we could not find any evidence for patients with endometriosis being of higher risk for elevated uNK cells. Endometriosis and increased uNK cells therefore seem to not be interdependent. In addition, we could not detect any statistical evidence that elevated levels of uNK cells or PC are a risk factor for RIF or RPL.

To summarize the data, analyzing uNK cells does not support a nonsurgical diagnosis of endometriosis whereas the analysis of uterine PC by endometrial scratching might be beneficial regarding the diagnosis of nonsurgically confirmed endometriosis and; therefore, improve the treatment of infertility patients with endometriosis in the clinical routine. The CE seems to be an above-average frequent concomitant of endometriosis.

## Data Availability

The datasets and code that support the findings of this study are available from the corresponding author, DM Baston-Büst, upon reasonable request.
